# Supplementary figures and images for: Quantification of dengue virus specific T cell responses and correlation with viral load and clinical disease severity in acute dengue infection
Source: PLoS Negl Trop Dis. 2018 Oct 1;12(10):e0006540. doi: 10.1371/journal.pntd.0006540 (PMC6181435; doi:10.1371/journal.pntd.0006540)

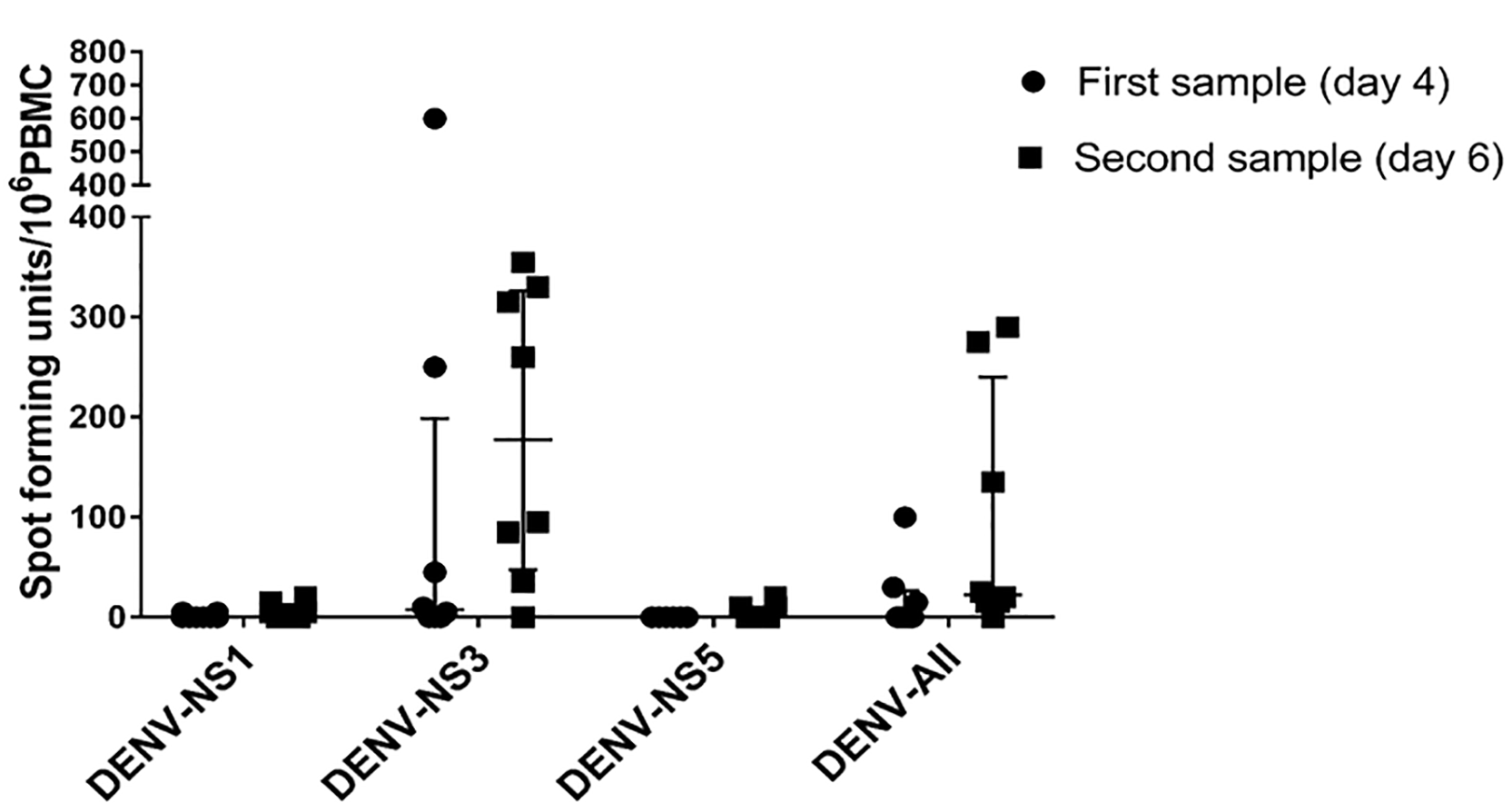

Supplement: S1 Fig — (TIF) [file pntd.0006540.s001.tif]

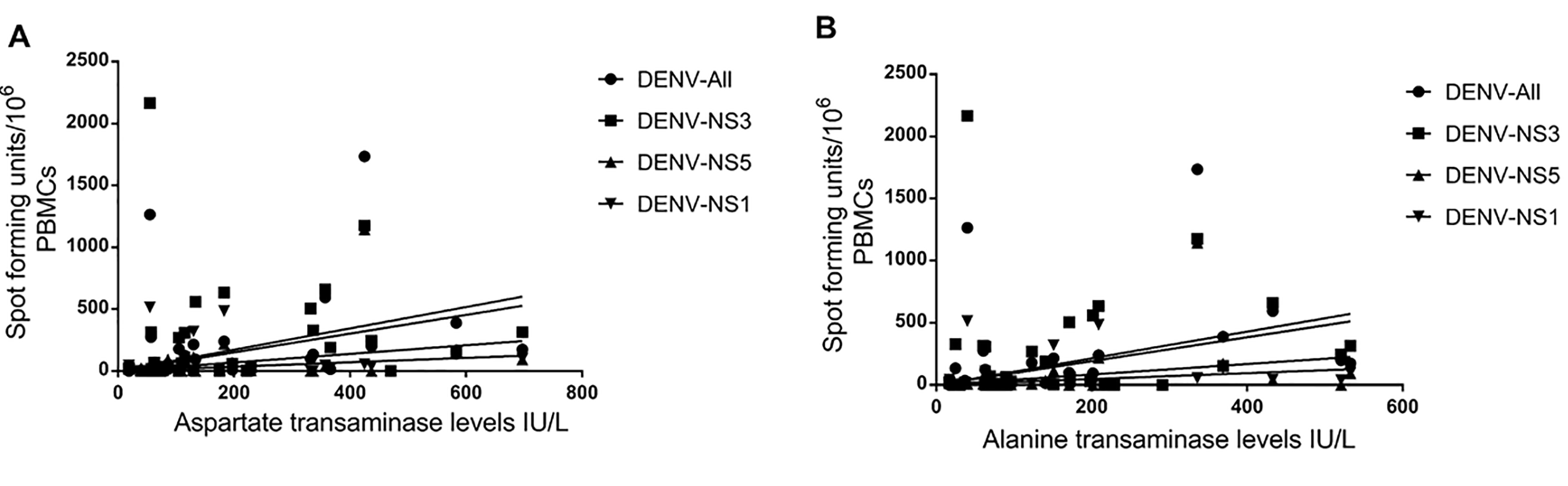

Supplement: S2 Fig — Association of DENV specific NS1, NS3, NS5 and All overlapping peptide responses in patients with acute dengue (n = 74) with the highest recorded aspartate transaminase level (NS1 Spearnman’s r = 0.15, p = 0.4; NS3 Spearman’s r = 0.34, p = 0.05; NS5 Spearman’s r = 0.16, p = 0.37; All Spearman’s r = 0.33, p = 0.06) (A) and alanine transaminase level (NS1 Spearman’s r = 12, p = 0.5; NS3 Spearman’s r = 0.25, p = 0.15; NS5 Spearman’s r = 0.19, p = 0.29; All Spearman’s r = 0.31, p = 0.08) (B). (TIF) [file pntd.0006540.s002.tif]

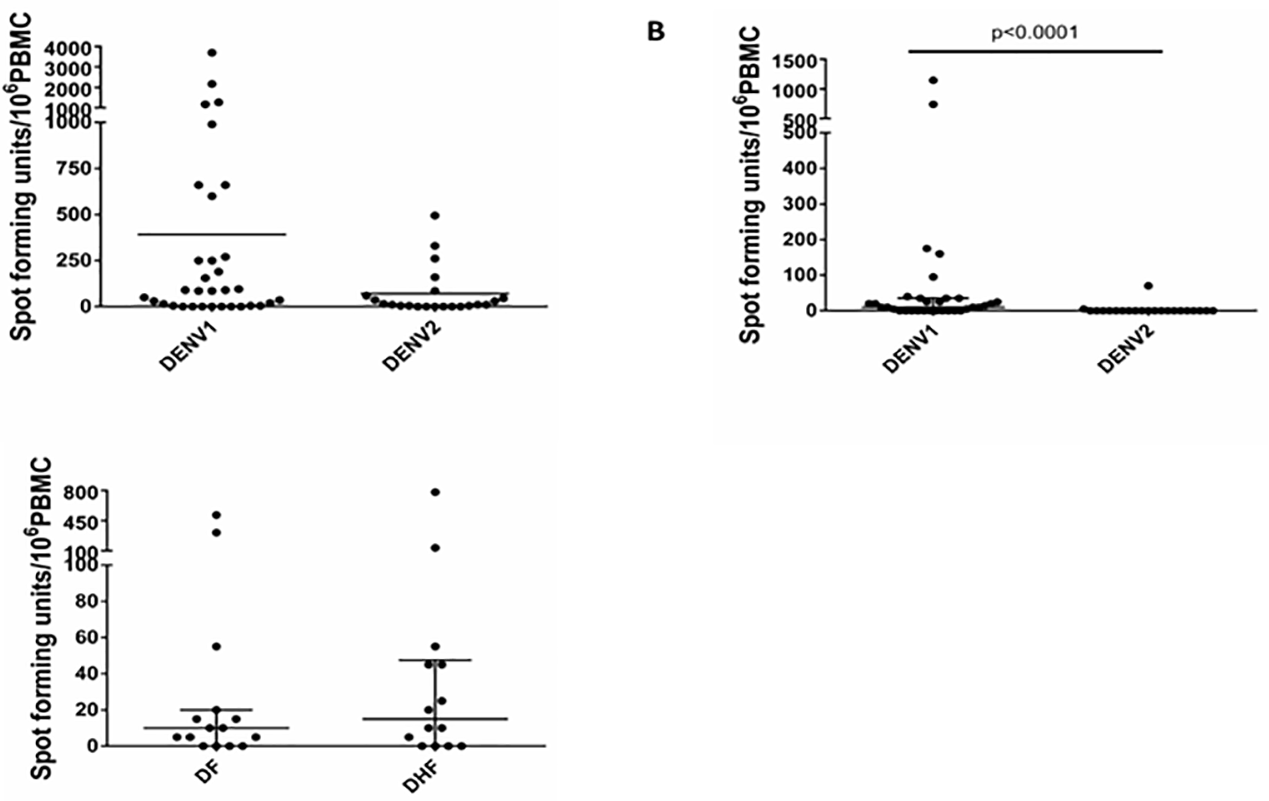

Supplement: S3 Fig — (A) IFNγ ELISpot responses were assessed in patients with acute DENV1 (n = 30) and acute DENV2 (n = 19) to NS3 overlapping peptides derived from DENV3 (B) IFNγ ELISpot responses were assessed in patients with acute DENV1 (n = 30) and acute DENV2 (n = 19) to NS5 overlapping peptides derived from DENV2 (C) IFNγ ELISpot responses were assessed in patients with acute DENV1 resulting in DF (n = 16) or DHF (n = 14) for NS1 overlapping peptides derived from DENV1. (TIF) [file pntd.0006540.s003.tif]

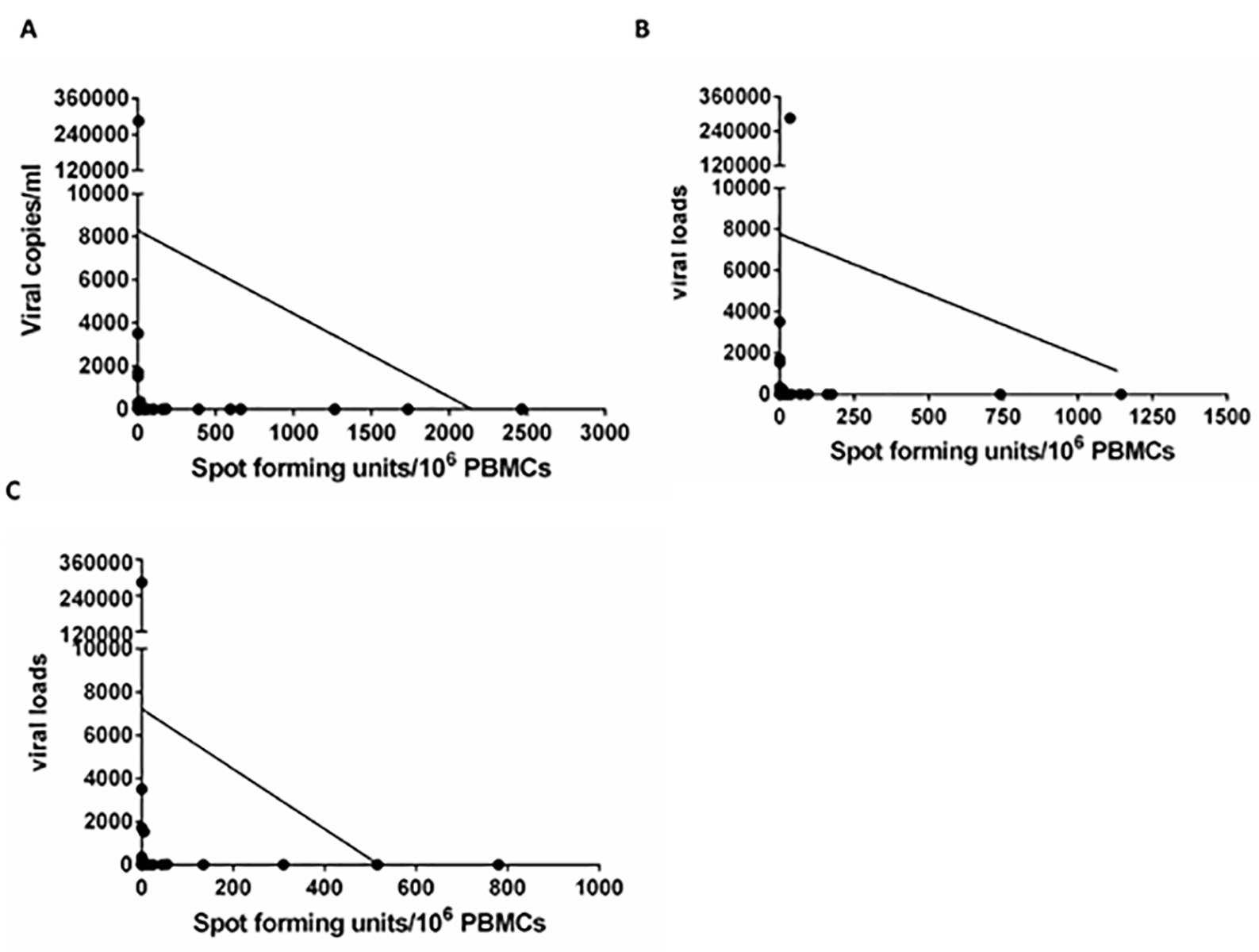

Supplement: S4 Fig — (A) Correlation between DENV All-Specific T cell responses and degree of viraemia (Spearman’s r = -0.38, p = 0.004). (B) Correlation between DENV NS5-Specific T cell responses and degree of viraemia (Spearman’s r = -0.28, p = 0.04). (C) Correlation between DENV NS1-Specific T cell responses and degree of viraemia (Spearman’s r = -0.31, p = 0.02). (TIF) [file pntd.0006540.s004.tif]
